# Detection of critical cerebral desaturation thresholds by three regional oximeters during hypoxia: a pilot study in healthy volunteers

**DOI:** 10.1186/s12871-016-0298-7

**Published:** 2017-01-13

**Authors:** Kerry L. Tomlin, Anna-Maria Neitenbach, Ulf Borg

**Affiliations:** Department of Medical Affairs, Patient Monitoring & Recovery, Medtronic, 6135 Gunbarrel Ave, Boulder, CO 80301 USA

**Keywords:** Regional oximetry, Cerebral oximetry, Near infrared spectroscopy, Cerebral desaturation, Hypoxia

## Abstract

**Background:**

Regional oximetry is increasingly used to monitor post-extraction oxygen status of the brain during surgical procedures where hemodynamic fluctuations are expected. Particularly in cardiac surgery, clinicians employ an interventional algorithm to restore baseline regional oxygen saturation (rSO_2_) when a patient reaches a critical desaturation threshold. Evidence suggests that monitoring cardiac surgery patients and intervening to maintain rSO_2_ can improve postoperative outcomes; however, evidence generated with one manufacturer’s device may not be applicable to others. We hypothesized that regional oximeters from different manufacturers respond uniquely to changes in oxygen saturation in healthy volunteers.

**Methods:**

Three devices were tested: INVOS™ 5100C (Medtronic), EQUANOX™ 7600 (Nonin), and FORE-SIGHT™ (CASMED) monitors. We divided ten healthy subjects into two cohorts wearing a single sensor each from INVOS and EQUANOX (*n* = 6), or INVOS and FORE-SIGHT (*n* = 4). We induced and reversed hypoxia by adjusting the fraction of inspired oxygen. We calculated the magnitude of absolute rSO_2_ change and rate of rSO_2_ change during desaturation and resaturation, and determined if and when each device reached a critical interventional rSO_2_ threshold during hypoxia.

**Results:**

All devices responded to changes in oxygen directionally as expected. The median absolute rSO_2_ change and the rate of rSO_2_ change was significantly greater during desaturation and resaturation for INVOS compared with EQUANOX (*P* = 0.04). A similar but nonsignificant trend was observed for INVOS compared with FORE-SIGHT; our study was underpowered to definitively conclude there was no difference. A 10% relative decrease in rSO_2_ during desaturation was detected by all three devices across the ten subjects. INVOS met a 20% relative decrease threshold in all subjects of both cohorts, compared to 1 with EQUANOX and 2 with FORE-SIGHT. Neither EQUANOX nor FORE-SIGHT reached a 50% absolute rSO_2_ threshold compared with 4 and 3 subjects in each cohort with INVOS, respectively.

**Conclusions:**

Significant differences exist between the devices in how they respond to changes in oxygen saturation in healthy volunteers. We suggest caution when applying evidence generated with one manufacturer’s device to all devices.

**Electronic supplementary material:**

The online version of this article (doi:10.1186/s12871-016-0298-7) contains supplementary material, which is available to authorized users.

## Background

A recent publication reported that surveyed cardiac anesthesiologists and perfusionists view regional oximetry as useful or essential for non-invasive monitoring of cerebral oxygen status during surgery [1]. Rapidly changing hemodynamic conditions can cause cerebral desaturation during cardiac, arthroscopic shoulder, major abdominal, and total knee replacement surgeries [2–5]. Originally published in 2007, clinicians continue to refine an interventional algorithm for maintaining cerebral oxygen saturation values (rSO_2_) close to baseline during cardiac surgery by manipulating oxygen supply and demand [6, 7]. Cerebral desaturation—often defined as a 20% relative decrease from baseline or an absolute rSO_2_ value of 50%—may trigger clinicians to consider a range of possible interventions to restore oxygen saturation, such as ruling out mechanical obstructions or increasing mean arterial pressure [7]. Several cardiac studies suggest postoperative outcomes may improve when cerebral oxygen saturation is monitored and desaturation episodes are recognized and reversed, compared to no or blinded monitoring [8–13].

We propose that published evidence generated with one manufacturer’s regional oximeter is unique to that device since they may reach an interventional threshold at different times or not at all. Each manufacturer uses a proprietary algorithm, as well as different emitter/detector spacing, number of wavelengths, and light source. We hypothesize that regional oximeters will react differently to clinically challenging situations where rSO_2_ is fluctuating.

To test our hypothesis, we subjected healthy volunteers to two cycles of desaturation and resaturation to characterize the differences in absolute rSO_2_ change and rate of rSO_2_ change between three FDA-cleared regional oximeters: INVOS™ 5100C (Medtronic), EQUANOX™ 7600 (Nonin), and FORE-SIGHT™ (CASMED) monitors. Changes in rSO_2_ were compared with changes in oxygen saturation as measured by pulse oximetry. Subjects wore one sensor each from two devices. We also took a unique approach from other comparative studies by tracking the time in which the devices reached critical rSO_2_ thresholds and whether the thresholds were met by one or both devices in individual subjects.

## Methods

The Western Institutional Review Board (Puyallup, WA) approved an overarching protocol for a study of pulse oximetry devices, of which this was studied under. Written informed consent was obtained prior to participation. Twelve healthy subjects were enrolled from an existing hypoxia research pool and studied at the Medtronic Respiratory & Monitoring Solutions clinical laboratory (Boulder, CO) from April 16 to 18, 2013. Two subjects were withdrawn: one failed screening, and the other experienced tachycardia and anxiety during the study. The study pool included males and non-pregnant or -lactating females, 18 to 50 years of age, from various racial and ethnic backgrounds, who tolerated hypoxia in previous studies. Skin pigmentation was rated as very light, olive, dark olive, or extremely dark.

Subjects reclined to approximately 20° in the supine position with legs elevated. Peripheral capillary oxygen saturation (SpO_2_) was monitored using the Nellcor N600x™ and Max-A™ sensors (Medtronic, Dublin, Ireland) on the middle and index fingers. Standard monitoring included continuous electrocardiography, noninvasive blood pressure, end-tidal carbon dioxide, and respiration rate.

**Fig. 1:**
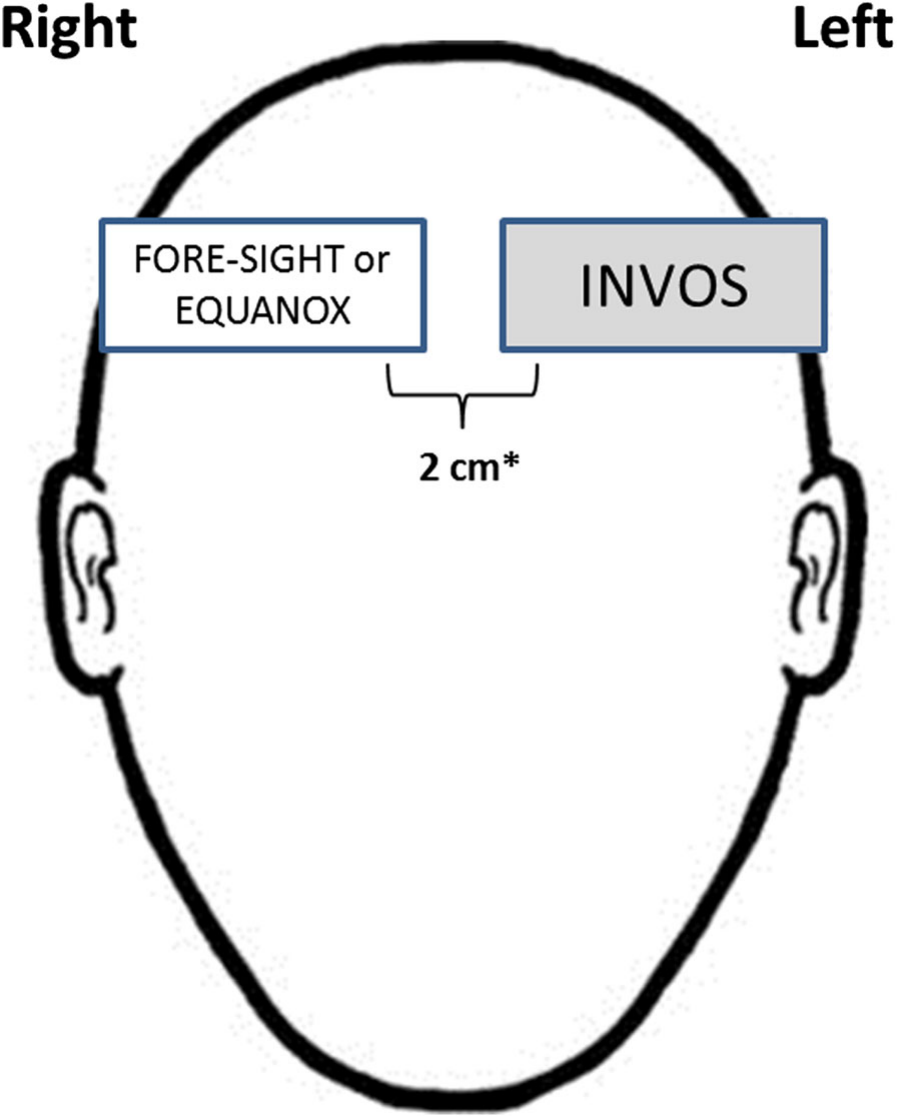
Placement of INVOS, EQUANOX, and FORE-SIGHT sensors on healthy human volunteers. * 2 cm separation distance was not possible in all subjects

We tested three FDA-cleared regional oximeters: INVOS 5100C (Medtronic, Dublin, Ireland), EQUANOX 7600 (Nonin Medical, Plymouth, MN), and FORE-SIGHT (CASMED, Branford, CT). All devices report regional oxygen saturation (rSO_2_) of the tissue beneath the sensor based on near infrared spectroscopy, the technical aspects of which are reported elsewhere [14, 15]. Two monitors were tested per subject with one INVOS sensor (SAFB-SM) on the left side of the forehead, and either an EQUANOX (8000CA or 8004CA) or FORE-SIGHT (standard large, medium, or small) sensor on the right, placed 2 cm apart if possible (Fig. 1). Six were studied with INVOS and EQUANOX, and four with INVOS and FORE-SIGHT.

**Fig. 2:**
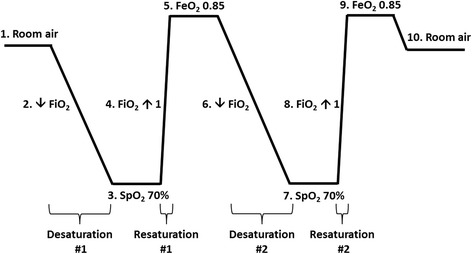
Method for inducing desaturation and resaturation in healthy subjects. Desaturation was induced by adjusting the gas mixture of oxygen and nitrogen through a facemask in healthy subjects. (1) Subjects breathed room air until a stable baseline rSO_2_ was achieved. (2–3) FiO_2_ was titrated down slowly to achieve a stable plateau SpO_2_ of approximately 70%. (4–5) FiO_2_ was increased instantly to 1 to achieve a stable FeO_2_ of 0.85. Steps (6) through (9) represent a second desaturation and resaturation cycle, after which (10) the subjects were returned to room air

Subjects were fitted with a gas delivery mask, through which the anesthesiologist adjusted the fraction of inspired oxygen (FiO_2_) using an oxygen-nitrogen gas mixer. The hypoxia protocol used meets the guideline defined in the ISO standard 80601-2-61:2011 for non-invasive laboratory testing on healthy volunteers for pulse oximetry, and is outlined in Fig. 2. (1) The initial baseline readings were established while breathing room air through the mask. (2) The FiO_2_ was decreased, based on the subject’s tolerance, to achieve an SpO_2_ reading of approximately 70%. (3) SpO_2_ was maintained at 70% for at least 60 s after the reading plateaued, up to 5 min. (4) The FiO_2_ was instantly increased to 1.0 to achieve a fraction of exhaled oxygen concentration (FeO_2_) of at least 0.85. (5–9) Plateau measurements were collected, and the desaturation cycle was repeated. (10) The mask was removed, and data collection continued for 5 min with the subject breathing room air. The anesthesiologist and lab clinician monitored vital signs and subject comfort throughout the experiment. Digital trending data from all monitors were collected and time-stamped.

Statistical analyses were performed using Minitab 17 (Minitab, Inc, Sate College, PA). Baseline rSO_2_ and absolute percent changes in SpO_2_ and rSO_2_ were graphed as individual subject data and the median for each device within the cohort. Rates of absolute oxygen saturation change (% per minute) were reported as the median and 95% confidence interval (CI) for each device within the cohort. Given the small sample size, normality was not assumed and the Wilcoxon Signed-Rank test for paired samples was used to assess significant differences in baseline rSO_2_ values, absolute rSO_2_ changes, and rates of rSO_2_ change between the devices within each cohort. Significant differences between devices in detecting critical rSO_2_ thresholds were assessed using the exact binomial test. A *P* value of <0.05 was considered statistically significant for all analyses.

## Results

We performed two rounds of desaturation and resaturation in ten healthy human subjects. The INVOS/EQUANOX and INVOS/FORE-SIGHT cohorts were similar in subject characteristics and gender distributions (Table 1). We included Caucasian (70%) and Asian (30%) subjects with a range of skin pigmentation from very light to dark olive; no African Americans were available to participate.

**Table 1:** Descriptive characteristics of study participants, mean ± SD or *n* (%); *N* = 10

Characteristic	INVOS/EQUANOX (*n* = 6)	INVOS/FORESIGHT (*n* = 4)
Age (yrs)	35.2 ± 10.2	31.8 ± 7.9
Male	3 (50%)	2 (50%)
Race/ethnicity		
Asian	2 (33%)	1 (25%)
Caucasian	4 (67%)	3 (75%)
Skin pigmentation		
Very light	4 (68%)	3 (75%)
Olive	1 (16%)	0 (0%)
Dark olive	1 (16%)	1 (25%)
Height (cm)	172.6 ± 4.7	176.6 ± 11.5
Weight (kg)^a^	75.1 ± 20.6	68.3 ± 7.1

^a^Weight was unavailable for one participant

### Baseline rSO_2_ prior to desaturation

**Fig. 3:**
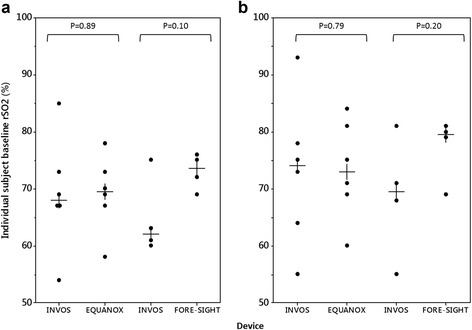
Individual baseline and median rSO_2_ values prior to desaturation #1 (**a**) and #2 (**b**). INVOS demonstrated a wider spread of individual baseline values than both EQUANOX and FORE-SIGHT. Although the median FORE-SIGHT baseline rSO_2_ was consistently higher than INVOS by approximately 10%, the differences were not statistically significant (two-tailed Wilcoxon Signed-Rank test for paired samples)

We plotted individual baseline rSO_2_ and median rSO_2_ values to visually compare the range and central tendency for each device (Fig. 3). The median baseline rSO_2_ prior to desaturation trended higher for FORE-SIGHT by approximately 10 percentage points compared with INVOS, although the differences were not statistically significant. Median INVOS and EQUANOX baseline rSO_2_ values differed by less than 2 percentage points. INVOS reported a wider range of individual baseline rSO_2_ values (54–93%) compared to EQUANOX (58–84%). FORE-SIGHT reported a narrower range of baseline values (69–81%) than both INVOS and EQUANOX.

### Absolute % change in rSO_2_ during desaturation and resaturation

**Fig. 4:**
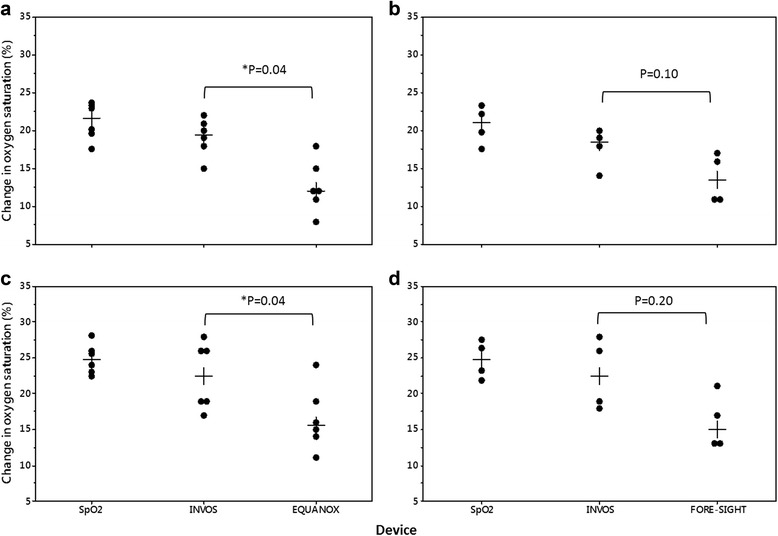
Individual absolute and median rSO_2_ changes during desaturation #1 (**a**, **b**) and #2 (**c**, **d**). Absolute changes in SpO_2_, representing global saturation, are shown for comparison. Median absolute change in rSO_2_ reported by INVOS during desaturation #1 and #2 were significantly greater than that of EQUANOX. Although FORE-SIGHT reported a smaller median absolute change during desaturation #1 and #2 compared with INVOS, the differences were not statistically significant (two-tailed Wilcoxon Signed-Rank test for paired samples). *Indicates statistical significance

**Fig. 5:**
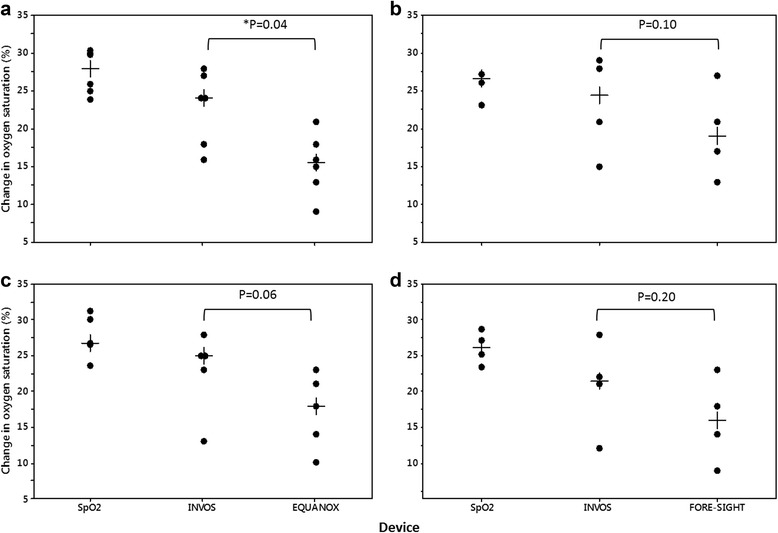
Individual absolute and median rSO_2_ changes during resaturation #1 (**a**, **b**) and #2 (**c**, **d**). Absolute changes in SpO_2_, representing global saturation, are shown for comparison. Median absolute change in rSO_2_ reported by INVOS during resaturation #1 was significantly greater than that of EQUANOX. Although FORE-SIGHT reported a smaller median absolute change during resaturation #1 and #2 compared with INVOS, the differences were not statistically significant (two-tailed Wilcoxon Signed-Rank test for paired samples). Data are missing for one INVOX/EQUANOX subject in graph C. *Indicates statistical significance

INVOS reported a significantly greater median absolute percent rSO_2_ change during both desaturation cycles compared with EQUANOX (*P* = 0.04), with a trend towards a greater change compared with FORE-SIGHT (*P* = 0.10) (Fig. 4). Although we did not compare the values statistically, the absolute percent change reported by INVOS (19.5–22.5%) followed closer in magnitude to the absolute changes in SpO_2_ (21.0–24.9%) compared with EQUANOX (12.0–15.5%) and FORE-SIGHT (13.5–15.0%). We discovered similar results during the two resaturation cycles, although the difference in absolute percent change in rSO_2_ for INVOS compared with EQUANOX during resaturation #2 only trended towards significance (*P* = 0.06) (Fig. 5).

### Rate of rSO_2_ change during desaturation and resaturation

We report the rates of rSO_2_ change during desaturation for each of the devices in Table 2, with SpO_2_ change shown for comparison. For both desaturation cycles, INVOS reported a significantly greater percent rSO_2_ change than EQUANOX (*P* = 0.04), with a trend towards a greater change than FORE-SIGHT (*P* = 0.10). The median rates of rSO_2_ change for INVOS (3.0–4.0%/min) followed closer in magnitude to rates of SpO_2_ change (3.6–4.3%/min) than EQUANOX (2.3–3.0%/min) or FORE-SIGHT (2.2–2.4%/min).

**Table 2:** Median rates (95% CI) of absolute oxygen saturation change during desaturation

	SpO_2_	INVOS	EQUANOX	
INVOS/EQUANOX (*n* = 6)	%/min	%/min	%/min	*P* value
Desaturation #1	4.2 (2.7, 5.2)	3.5 (2.9, 4.3)	2.3 (1.7, 2.8)	0.04^a^
Desaturation #2	4.3 (3.5, 5.5)	4.0 (3.0, 4.7)	3.0 (2.0, 3.5)	0.04^a^
	SpO_2_	INVOS	FORE-SIGHT	
INVOS/FORE-SIGHT (*n* = 4)	%/min	%/min	%/min	*P* value
Desaturation #1	3.6 (2.9, 4.1)	3.0 (2.5, 3.5)	2.4 (1.8, 2.8)	0.10
Desaturation #2	3.7 (2.9, 5.0)	3.4 (2.4, 4.9)	2.2 (1.7, 4.0)	0.10

^a^Indicates statistical significance between devices (two-tailed Wilcoxon Signed-Rank test for paired samples)

The rate of rSO_2_ change during resaturation is reported in Table 3. INVOS reported a significantly greater rate of change compared with EQUANOX during resaturation #1 (*P* = 0.04), with a trend towards a greater rate during resaturation #2 (*P* = 0.06). Although numerically higher than FORE-SIGHT, the rates of rSO_2_ change for INVOS during both resaturation cycles were not statistically different. The median rates of rSO_2_ change for INVOS (9.6–15.9%/min) were closer in magnitude to rates of SpO_2_ change (11.75–19.1%/min) than EQUANOX (9.2–11.2%/min) or FORE-SIGHT (6.6–9.5%/min).

**Table 3:** Median rates (95% CI) of absolute oxygen saturation change during resaturation

	SpO_2_	INVOS	EQUANOX	
INVOS/EQUANOX (*n* = 6)	(%/min)	(%/min)	(%/min)	*P* value
Resaturation #1	19.1 (13.8, 25.7)	15.9 (12.5, 18.5)	11.2 (7.0, 13.4)	0.04^a^
Resaturation #2	15.3 (11.6, 21.9)	12.7 (8.5, 17.7)	9.2 (5.2, 14.5)	0.06
	SpO_2_	INVOS	FORE-SIGHT	
INVOS/FORE-SIGHT (*n* = 4)	(%/min)	(%/min)	(%/min)	*P* value
Resaturation #1	12.9 (10.1, 18.8)	10.6 (8.1, 19.3)	9.5 (6.5, 14.4)	0.10
Resaturation #2	11.7 (10.4, 13.1)	9.6 (5.8, 12.5)	6.6 (4.4, 12.0)	0.20

Data are unavailable for one INVOX/EQUANOX subject. ^a^ Indicates statistical significance between devices (two-tailed Wilcoxon Signed-Rank test for paired samples)

### Critical desaturation thresholds

We reviewed the rSO_2_ values from desaturation #1 for the following thresholds: 1) 10% relative change from baseline, 2) 20% relative change from baseline, and 3) an absolute value of 50%. For each subject, we determined whether the devices reached the threshold, and the mean time difference when the threshold was met by both devices.

We considered a 10% relative change from baseline as an early indicator that cerebral oxygen saturation is decreasing and may require early intervention [2]. All three devices reached the 10% relative change in all subjects of both cohorts. On average, INVOS reached the 10% threshold 28 s earlier than EQUANOX, and 43 s earlier than FORE-SIGHT.

Table 4 shows the number of subjects in which only one monitor for each pair detected a critical drop in rSO_2_. In the INVOS/EQUANOX cohort, INVOS met the 20% relative decrease for all six subjects in the cohort. In five of six subjects, EQUANOX did not detect a 20% relative decrease. There were no subjects for whom EQUANOX reached the threshold and INVOS did not, and although numerically different, the results only trended towards statistical significance (*P* = 0.06). We found similar results with the INVOS/FORE-SIGHT cohort, where INVOS met the 20% threshold in all four subjects compared with two subjects with FORE-SIGHT. There were no subjects for whom FORE-SIGHT detected a 20% relative change when INVOS did not. The difference was not statistically significant.

**Table 4:** Detection of critical rSO_2_ thresholds during the first desaturation cycle (*n*, %)

	INVOS: YES EQUANOX: NO *n* = 6	INVOS: NO EQUANOX: YES *n* = 6	*P* value	INVOS: YES FORE-SIGHT: NO *n* = 4	INVOS: NO FORE-SIGHT: YES *n* = 4	*P* value
20% relative rSO_2_ decrease from baseline met	5 (83%)	0	0.06	2 (50%)	0	0.50
50% absolute rSO_2_ threshold met	4 (67%)	0	0.06	3 (75%)	0	0.25

Data were not statistically significant (exact binomial test)

When we used an absolute threshold of 50%, INVOS reached the threshold in four of six subjects, whereas EQUANOX did not in any subjects, with a trend towards statistical significance (*P* = 0.06). In the INVOS/FORE-SIGHT cohort, INVOS reached the threshold in three of four subjects, while FORE-SIGHT did not in any subjects. Again, the results were not statistically significant.

## Discussion

All three devices reported changes in rSO_2_ during the course of desaturation and resaturation. We found that the median absolute change in rSO_2_, and the rate of rSO_2_ change per minute, was significantly greater with INVOS compared with EQUANOX during desaturation. The magnitude and rate of change was numerically greater with INVOS compared to FORE-SIGHT, but the differences were not statistically significant. When we reversed desaturation, we found similar significant differences in the magnitude and rate of change between INVOS and EQUANOX, with a trend towards significance between INVOS and FORE-SIGHT.

The most interesting finding was the discordance in detecting critical desaturation thresholds during desaturation when comparing devices on the same subject. All three technologies detected a minimum 10% relative decrease in rSO_2_ from baseline in all subjects; EQUANOX and FORE-SIGHT reached the threshold on average 28 to 43 s after INVOS, respectively. INVOS detected a 20% relative decrease in rSO_2_ in all subjects of both cohorts, compared to one with EQUANOX, and two subjects with FORE-SIGHT. Neither EQUANOX nor FORE-SIGHT met the 50% absolute rSO_2_ threshold in any subjects, compared to four and three subjects with INVOS, respectively. While our findings lacked statistical significance, they may have important clinical implications.

The randomized, controlled evidence reporting improved postoperative outcomes compared with no monitoring is based on detecting a critical drop in rSO_2_ in the monitored subjects that the clinician observes and intervenes to reverse [4, 8–12]. These studies usually cite a threshold of either a 20 to 25% relative decrease from baseline, or absolute value of 50 to 60%. Our study has shown that regardless of whether a relative or absolute threshold is used, disparities may exist between devices in detecting desaturation events. These disparities may result in clinicians intervening, for example, earlier and/or more often when using one device compared to another. A clinician may employ cerebral oximetry to improve outcomes and see different results from published trials if the same device is not used. We make no judgment about which device is “correct,” simply that they are different from one another.

Device design may contribute to the differences in reporting rSO_2_ during changes in cerebral oxygen saturation. Manufacturers make unique assumptions of arterial versus venous contribution of the tissue under the sensor: 25/75 for INVOS, and 30/70 for EQUANOX and FORE-SIGHT. Each device uses a distinct proprietary algorithm. INVOS 5100C has a 2-wavelength LED light source in the sensor, compared with a 3-wavelength LED in EQUANOX 7600 and a 4-wavelength laser in FORE-SIGHT. The devices also sample different tissue depths due to individual sensor/detector spacing.

Our findings corroborate those published from previous device comparisons in both healthy subjects and surgical patients, with some exceptions. Fellahi and colleagues found a greater percent maximum difference from baseline with INVOS compared with EQUANOX in healthy subjects during leg vascular occlusion tests [16]. In 42 off-pump coronary artery bypass surgery patients, Moerman and colleagues reported a greater area under the curve for INVOS during desaturation compared to FORE-SIGHT (−68%/s vs −39%/s) and only a weak correlation in rSO_2_ values between the two devices (*r* = 0.31) [17]. In contrast, Closhen and colleagues found similar changes in rSO_2_ between INVOS and FORE-SIGHT in 35 patients moved into the beach-chair position for arthroscopic shoulder surgery, although the absolute change in rSO_2_ for both devices during repositioning was minimal (10%) [18]. The authors reported a stronger correlation in rSO_2_ values between the two devices (*r* = 0.68) than Moerman and colleagues. Both Moerman and Fellahi reported a wider range of values from INVOS compared with FORE-SIGHT and EQUANOX, respectively [16, 17].

Fellahi and colleagues also found significantly greater rates of rSO_2_ change during desaturation (3.65%/min vs 2.36%/min) and resaturation (30.4 vs 16.8%/min) for INVOS compared with EQUANOX, similar to our own findings [16]. But Hyttel-Sorensen and colleagues reported a steeper desaturation slope with EQUANOX during arm vascular occlusion in 10 healthy volunteers compared with INVOS and FORE-SIGHT [19].

Few studies compared how and when different devices detect clinically relevant desaturation events. Pisano et al., compared devices in cardiac surgery and reported that INVOS detected 20 significant cerebral desaturation events in four of ten patients, compared with three events in one patient for EQUANOX (the same patient in which INVOS detected five events) [20]. Unlike our study, two sensors per device were placed bilaterally, with one sensor pair placed above the other on the forehead. We cannot compare these results to ours since Pisano et al., recorded rSO_2_ from only one device at a time due to interference, and as such would not have detected the same desaturation events. In Closhen’s beach-chair study, INVOS reported one event of rSO_2_ less than 50% during positioning that was not reported by FORE-SIGHT [18]. In a multicenter, randomized, controlled study of high-risk cardiac surgery patients where all three devices were used, Deschamps et al., found that INVOS detected on average 2.3 desaturation events per patient, compared with 1.7 for EQUANOX, and 1.5 for FORE-SIGHT, although only one device was used per patient so there were no direct comparisons of detecting the same event [2].

We point out several important limitations in our study for consideration. We were not able to place the sensors 2 cm apart on all subjects, and we did not investigate whether there was interference between the devices at close distances. We did not calculate a sample size a priori based on a predetermined difference between devices in either the magnitude or rate of rSO_2_ change. In cases where we observed no significant difference between devices, we are unsure if there truly was no difference, or if our study lacked sufficient power. We tend to believe the latter, particularly with the 4-subject INVOS/FORE-SIGHT cohort.

Also, we suggest interpreting differences in rSO_2_ rates of change during desaturation in our study with caution; the anesthesiologist titrated FiO_2_ gradually as the subject tolerated it, which may introduce between-subject variation. Baseline rSO_2_ reported by INVOS was notably lower in some subjects than EQUANOX and FORE-SIGHT, potentially influencing whether a device reached the 50% absolute rSO_2_ threshold during desaturation. Also, unlike two of the papers cited here, we used each device unilaterally [17, 20]. We concede that one device could have reached a threshold earlier than (or in the absence of) another due to hemispheric differences. Finally, we did not directly sample arterial and venous blood for calculating a weighted saturation as a reference to compare with rSO_2_ values. As such, we cannot comment on which device best represented cerebral oxygen status. But despite the small nature of our pilot study, the hypoxia protocol reported here is repeatable and widely used for validating regional oximeters for agency approval. We generated intriguing preliminary results to inform future hypotheses and hopefully generate interest in larger, more comprehensive device comparisons.

With these preliminary results, one might consider whether clinical evidence generated in one manufacturer’s regional oximetry device can be broadly applied to similar devices from other manufacturers. The three devices tested in our study reached critical interventional rSO_2_ thresholds inconsistently in the same subject. Although the differences between devices in detecting critical rSO_2_ thresholds were not statistically significant, we argue that the results may have clinical significance. A larger study with adequate power may clarify our findings.

## Conclusions

To the best of our knowledge, our report is the first to show differences between INVOS and both FORE-SIGHT and EQUANOX in detecting the same desaturation event when subjects are monitored simultaneously. With this knowledge, one should take care in broadly applying evidence of improved patient outcomes to all devices when a single manufacturer’s device is used in a study. Larger studies in clinical settings are required to investigate the clinical impact of this finding.

## Additional files

Additional file 1: Raw data subject #2234. (XLSX 107 kb)
Additional file 2: Raw data subject #1228. (XLSX 123 kb)
Additional file 3: Raw data subject #1654. (XLSX 174 kb)
Additional file 4: Raw data subject #1657. (XLSX 122 kb)
Additional file 5: Raw data subject #1667. (XLSX 139 kb)
Additional file 6: Raw data subject #1791. (XLSX 116 kb)
Additional file 7: Raw data subject #1806. (XLSX 128 kb)
Additional file 8: Raw data subject #1824. (XLSX 118 kb)
Additional file 9: Raw data subject #2142. (XLSX 124 kb)
Additional file 10: Raw data subject #2174. (XLSX 112 kb)
Additional file 11: Tabulated data from all subjects. (XLSX 62 kb)

## Abbreviations

FeO_2_: Fraction of exhaled oxygen
FiO_2_: Fraction of inhaled oxygen
rSO_2_: Cerebral oxygen saturation
SpO_2_: Peripheral capillary oxygen saturation
